# Assessing the impact of revegetation and weed control on urban sensitive bird species

**DOI:** 10.1002/ece3.2960

**Published:** 2017-05-02

**Authors:** Carla L. Archibald, Matthew McKinney, Karen Mustin, Danielle F. Shanahan, Hugh P. Possingham

**Affiliations:** ^1^ Centre for Biodiversity and Conservation ScienceSchool of Biological SciencesThe University of QueenslandBrisbaneQldAustralia; ^2^ School of Life SciencesImperial College of LondonAscot SL5 7PY, UKUK

**Keywords:** Bayesian, hierarchical community model, urban conservation, urban restoration, urban sensitive species

## Abstract

Nature in cities is concentrated in urban green spaces, which are key areas for urban biodiversity and also important areas to connect people with nature. To conserve urban biodiversity within these natural refugia, habitat restoration such as weed control and revegetation is often implemented. These actions are expected to benefit biodiversity, although species known to be affected by urbanization may not be interacting with restoration in the ways we anticipate. In this study, we use a case study to explore how urban restoration activities impact different bird species. Birds were grouped into urban sensitivity categories and species abundance, and richness was then calculated using a hierarchical species community model for individual species responses, with “urban class” used as the hierarchical parameter. We highlight variable responses of birds to revegetation and weed control based on their level of urban sensitivity. Revegetation of open grassy areas delivers significant bird conservation outcomes, but the effects of weed control are neutral or in some cases negative. Specifically, the species most reliant on remnant vegetation in cities seem to remain stable or decline in abundance in areas with weed control, which we suspect is the result of a simplification of the understorey. The literature reports mixed benefits of weed control between taxa and between locations. We recommend, in our case study site, that weed control be implemented in concert with replanting of native vegetation to provide the understory structure preferred by urban sensitive birds. Understanding the impacts of revegetation and weed control on different bird species is important information for practitioners to make restoration decisions about the allocation of funds for conservation action. This new knowledge can be used both for threatened species and invasive species management.

## 1 Introduction

Seventy percent of the global human population will live in urban areas by 2050 (United Nations 2014). This growth will trigger major urban expansion and, in many scenarios, provides a bleak outlook for biodiversity occupying natural areas within and surrounding cities. Green spaces within urban landscapes provide local refugia for species, particularly those more sensitive to urban activity (Fernández‐Juricic & Jokimäki, 2001; Ives et al., 2015). Preserving and restoring urban green spaces enables urban biodiversity to remain present within these ever changing environments.

Restoration within urban landscapes also provides a platform to promote a conservation ethic among the general population through increased exposure to biodiversity (Miller, 2005; Pyle, 1978). This is particularly important as people are becoming less connected to nature and biodiversity (Miller, 2005; Pyle, 1978). Birds may be particularly important species to connect people to nature as they are relatively conspicuous, and therefore can be easily observed by many people. Furthermore, there is evidence that knowledge of local avifauna positively influences how people feel about the green spaces they use. The connection people feel toward nature has also been shown to increase when species diversity of birds is highest (Caula, Hvenegaard, & Marty, 2009; Cox & Gaston, 2015; Hedblom et al., 2014), which in itself can be affected by the quality and quantity of green spaces within urban landscapes (Carbó‐Ramírez & Zuria, 2011; Huang et al., 2015; Imai & Nakashizuka, 2010; Shanahan, Possingham, & Martin, 2011; Strohbach, Lerman, & Warren, 2013).

The impacts of fragmentation and degradation due to urbanization on biodiversity have been documented widely (Venter et al., 2016; Zipkin, Dewan, & Royle, 2009), including in green spaces within urban areas (Fernández‐Juricic & Jokimäki, 2001). Bird species richness and abundance within the broader landscape context have been shown to be negatively affected by the loss and fragmentation of green spaces (Crooks, 2004; Shanahan, Possingham, et al., 2011; Zipkin et al., 2009). Therefore, restored urban greenspaces can benefit urban areas as well as the broader landscape, possibly by providing refugia for urban biodiversity (Fernández‐Juricic & Jokimäki, 2001; Shanahan, Miller, et al., 2011; Shanahan, Possingham, et al., 2011).

Urbanization can cause a shift in bird species relative abundance toward a system dominated by “urban adapters” and “urban exploiters” (Blair, 1996; Dearborn & Kark, 2010; Kark et al., 2007; Manfredo et al., 2016). These are those species that dominate highly urbanized surroundings. For example, in Australia species such as the Australian white ibis (*Threskiornis moluccus*), rock dove (*Columba livia*), house sparrow (*Passer domesticus*), and noisy miner (*Manorina melanocephala*), all successfully exploit the urban environment (Kark et al., 2007). In such systems, management actions may variably benefit or harm species depending on their urban sensitivity. It is therefore logical that managers of urban green spaces might wish to tailor their actions to benefit species whose abundances tend to be highest in undisturbed habitats, and that therefore tend to decline more strongly as a result of urbanization, here termed “urban sensitive species,” rather than species already well‐adapted to urban areas.

Two globally common restoration actions are the control of non‐native vegetation (hereafter referred to as “weed control,” and restoration of native vegetation to previously cleared areas (hereafter referred to as revegetation) (Brisbane City Council 2015; Marzluff & Ewing, 2001; National Landcare Programme 2016). Revegetation and weed control address persistent threats and pressures such as weed propagule pressure, disturbance, and species invasions that accompany intensive human land use (Heinrichs, 2015). These restoration activities are often assumed to yield ecological benefits, but when particular bird groups and species become the management target, this assumption can break down (Lampert et al., 2014). The effect of invasive weed management on native bird diversity has been heavily debated, and the benefits may vary depending on the species present. There is evidence that some weedy areas offer more food resources and nesting spaces than nonweedy areas, or that they act as refugia where no other suitable habitat is available (Gosper & Vivian‐Smith, 2009; Rogers & Chown, 2014). However, the benefits for some bird species are less clear, with no preference shown between weedy and nonweedy areas (Gan et al., 2009). Declines in bird species richness in some areas have also been attributed to weeds due to decreases in structural complexity and plant diversity (Aravind et al., 2010; Milton et al., 2007; Skórka, Lenda, & Tryjanowski, 2010).

While evidence from revegetation projects in nonurban locations shows that vegetation structure can greatly influence bird diversity outcomes (Lindenmayer et al., 2008, 2012; Munro et al., 2011), revegetation requires a substantial time investment, and it can take years for biodiversity benefits to be observed (Vesk & Nally, 2006). Both revegetation and invasive weed control are expensive, and the outcomes of individual projects are variable (Aravind et al., 2010; Barrett et al., 2008; Freeman, Catterall, & Freebody, 2015; Grman, Bassett, & Brudvig, 2013; Lindenmayer et al., 2012). Planning for the management of urban green spaces is further complicated by trade‐offs between the needs and wants of different stakeholder groups (Dearborn & Kark, 2010; Main, Roka, & Noss, 1999; McAlpine et al., 2016; Sol et al., 2014). There is a need to employ evidence‐based cost‐effective approaches to plan for green space management, thus providing better on‐the‐ground results for the available budget, and to take into account pros and cons of different management options (Jellinek et al., 2014; Lindenmayer et al., 2012).

Here, we explore the relative conservation benefit of two restoration strategies: revegetation of open‐mowed grass areas and weed control of invasive plant species in native forest patches. We propose this comparison between revegetation and weed control as these methods of vegetation restoration often compete for the same economic resources. Understanding how each restoration action impacts bird diversity will provide necessary insight for restoration strategy and decision making. When land managers engage in restoration activities to increase bird diversity, clear targets must be identified. For example, managers may want to identify actions that will likely increase the abundance of urban sensitive species, which tend to avoid urban areas and require more natural habitat to persist, as opposed to increasing abundances of urban exploitative species—those species that do well in highly modified, urban habitats. We expect to find that urban exploitative species, urban adaptable species, and urban sensitive species will respond differently to revegetation and weed control in urban areas. Specifically, we expect that: (1) urban exploiter species will be more abundant in disturbed than restored areas; (2) the abundance of urban adaptable species will not differ significantly between disturbed and restored areas; and (3) urban sensitive species will be more abundant in restored than disturbed habitats. We use a case study to evaluate the relative benefits of restoration actions in an urban setting. We highlight the importance of measuring the impact of restoration not only on all bird diversity, but rather species more sensitive to urban areas to maximize desired outcomes and avoid undesired outcomes. Identifying how urban sensitive species are impacted by different types of restoration directly relates to how successful the restoration action is in increasing important urban bird diversity.

## 2 Materials and Methods

### 2.1 Study area

**Figure 1:**
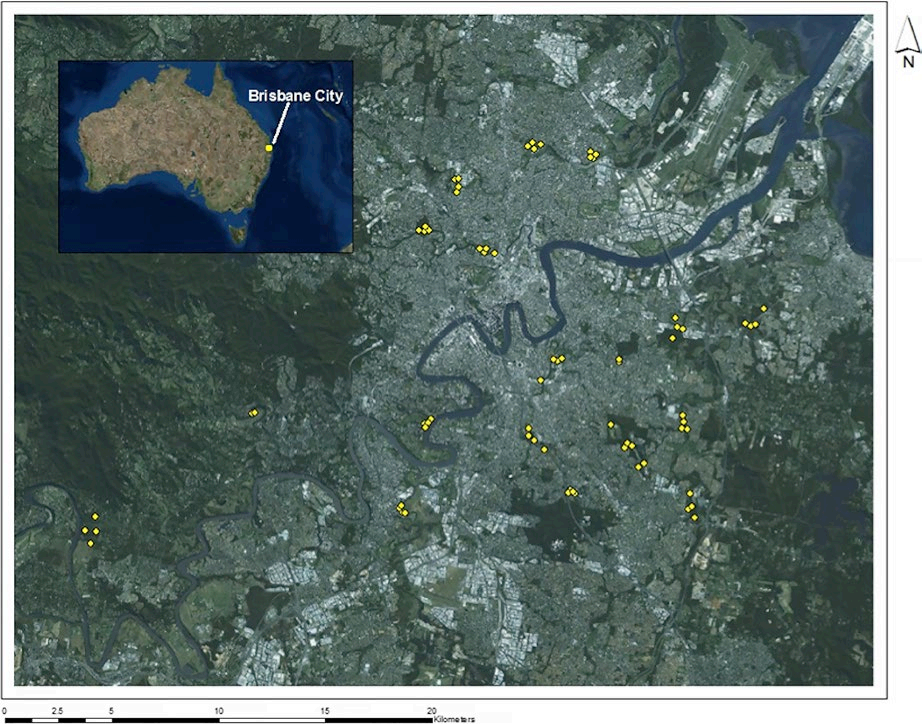
Points indicate the 70 sites surveyed around Brisbane, Queensland

The study was conducted at 19 blocks across Brisbane City (S 27.4679°, E 153.0278°) Queensland, Australia (Figure 1). The sites were all managed by local councils and community groups that have been historically implementing weed control and revegetation within the area over the past 25 years (Brisbane City Council 2015). Within each block, all four treatment types were present: revegetation, open mowed grass, forest sites with weeds, and forest sites without weeds.

### 2.2 Data collection

#### 2.2.1 Habitat data

Management history for each location was collected from project participants during a prior study (Shanahan, Miller, et al., 2011; Shanahan, Possingham, et al., 2011), and all 70 sites were surveyed for vegetation structure and composition. We used a space‐for‐time substitution method to evaluate the impact of each restoration action compared to its counterfactual (Pickett, 1989). Each revegetation site was paired with a nearby open‐mowed grass site of similar size as a counterfactual (*n* = 19) (Pickett, 1989). Data were collected in 19 revegetation sites that have been planted with eucalypts, acacias, and callistemons to form an open dry‐sclerophyll habitat. The understory was planted out with grasses and lomandras, or manicured into paths or garden beds with mulch and stones. To test for the effect of weed control, forest patches were assessed using a vegetation survey to determine invasive weed cover. Sites with weeds were identified as those containing >60% weed cover (*n* = 16), and sites without weeds were identified by containing <15% weed cover (*n* = 16). Three blocks failed to meet these criteria and were excluded within the weed control analysis. Forest sites both with and without weeds were predominantly dry‐sclerophyll eucalypt and acacia forests. Plant species that are invasive in Australia (“weeds”) present at the sites include lantana (*Lantana camara*) and Ochna (*Ochna serrulata*). All sites were between 0.5 and 2.5 ha, and sites in the same block did not vary by more than 0.5 ha. The area and boundary length were determined for each site, as well as for the larger vegetation patch within which each site is situated, using Google Earth. To calculate these metrics, the patch was defined to include any green area connected to the site.

#### 2.2.2 Bird data

All sites were visited on three occasions in the early morning (05:00 hr–08:30 hr) during September 2013 to February 2014. These dates correspond to the breeding season for most terrestrial birds in southeast Queensland. Sites were covered by walking variable paths during a 20‐min period, and all birds seen or heard within the site were recorded (Loyn, 1986). Water birds and birds that were not observed utilizing the site (e.g., flying overhead) were not included in the dataset for analysis, except for species which capture or search for their prey from the air (e.g., birds of prey and swallows).

### 2.3 Data analysis

#### 2.3.1 Definition of urban classes

The interaction between bird species and restoration were analyzed individually to highlight impacts of revegetation and weed control to all species that were detected within the case study site. In addition, to characterize variable responses of groups of birds to management, we assigned each bird species to an “urban class” (i.e., how likely a species is to be found in an urban setting): urban sensitive, urban adaptable, and urban exploitative. We independently assessed all birds observed during this study based on expert opinion, ecology, diet, breeding, and habitat information and assigned them an urban sensitivity category (Catterall et al., 2010; Garnett et al., 2014; Marchant et al., 1990; Sewell & Catteral, 1998; Sushinsky et al., 2013). These categories served not only as an ecological grouping scheme, but also as an objective driven grouping scheme, whereas urban sensitive birds could be treated as higher priority management targets. Tables S1 and S2 in the supplementary information display these allocations.

#### 2.3.2 Estimation of species richness and abundance

We used a Bayesian hierarchical community model to simultaneously estimate urban class‐specific response hyperparameters and species‐specific response parameters (Kery & Schaub, 2012; Pacifici et al., 2014; Riffell et al., 2015; Royle & Dorazio, 2008). This Bayesian analysis method is analogous to a mixed‐effects model, where species are random effects. However, the Bayesian hierarchical community model can directly estimate urban class‐level parameters, in the form of a posterior probability distribution, from species‐specific parameters. Additionally, uncommon (and therefore harder to observe) species modeling can be improved by using class‐specific hyperparameters and prior information (Zipkin et al., 2009). Therefore, we can make direct statements about the probability that a treatment was beneficial, both at the species and urban class‐level.

From the count data collected, we estimated latent (un‐observed) abundance for each species, accounting for the species detectability using *N*‐mixture models (Royle, 2004).

Over dispersion due to detection bias is common when working with bird data; therefore, we used a zero inflated Poisson mixture model to model species abundance (Kery & Schaub, 2012). In this type of model, species abundance is conditional on the additional species inclusion parameter *w*_*ij*_ for species *i* occurrence at site *j*. Here, *w*_*ij*_ was the outcome of a Bernoulli process with the probability ψ_*i*_:

(1) $$w_{ij}∼\text{Bernoulli}\;\left(\psi_{i}\right)$$

For all species *i,* the latent abundance *N*_*ij*_ was calculated for each site *j* assuming a Poisson distribution, conditional on inclusion:

(2) $$N_{ij}∼\text{Poisson}\;\left(w_{i}\cdot \lambda_{ij}\right)$$

where λ_*ij*_ represents variation in latent abundance for each species *i* at each site *j*. We then modeled variation in abundance on the log scale:

(3) $$\begin{array}{cc} \text{log}\;(\lambda_{ij})\;=\; & u1_{i}{\text{TRT}}_{j}\;+\;u2_{i}\;\left(1-{\text{TRT}}_{j}\right)\;+\;o1_{i}{\text{ORI}}_{j}\;+\;o2_{i}\;\left(1-{\text{ORI}}_{j}\right)\\ & \;+\;\alpha 1_{i}\text{area}1_{j}\;+\;\alpha 2_{i}\text{area}1_{j}{\text{TRT}}_{j}\;+\;\alpha 3_{i}{\text{TRT}}_{j}{\text{ORI}}_{j}\\ & +\;\alpha 4_{i}\text{TRT}\;\left(1-{\text{ORI}}_{j}\right) \end{array}$$

where *u*1_*i*_ and *u*2_*i*_ are the species‐specific parameters (for each species *i*) for “treated” (coded as 1 in TRT) and “untreated” (coded as 0 in TRT) sites *j*. In this model, “treated” can represent the case of a revegetated site, or a weed‐controlled site. For ease of parameterization, we paired sites with the same origin; sites with and without weeds were categorized as “forest” origin, and revegetated sites and open‐mowed grass sites were categorized as “grass” origin. *o*1_*i*_ and *o*2_*i*_ are the species‐specific parameters for origin of each site *j* (forest origin coded 1 in ORI; grass origin coded 0). We also included species‐specific parameters for patch area (α) at each site *j*, and the patch area × treatment interaction (α2) at each site *j*, and the treatment × origin interaction (α3 and α4) at each site *j*. We included the interaction for patch area × treatment to highlight whether the observed outcome of treatment was in anyway confounded by patch size. The interaction of treatment × origin highlights the observed impact of treatment under different management actions.

We modeled observed abundance *y*_*ijk*_ at each visit *k* as a binomial outcome with parameters of *n*_trials_ = latent abundance and *p*(success) = detection probability $p_{ijk}$:

(4) $$y_{ijk}∼\text{Binomial}\;\left(p_{ijk},\;N_{ij}\right)$$

We modeled the detection probability as a logit function of individual species (*v*1_*i*_), linear (β1_*i*_), and quadratic (β2_*i*_) effects for days since surveys began (DATE), and linear (β3_*i*_), and quadratic (β4_*i*_) effects of minutes since sunrise (TSR) for each survey at site *j* and visit *k*:

(5) $$\text{logit}\;\left(p_{ijk}\right)\;=\;v1_{i}\;+\;\beta 1_{i}{\text{DATE}}_{jk}\;+\;\beta 2_{i}{\text{DATE}}_{jk}^{2}\;+\;\beta 3_{i}{\text{TSR}}_{jk}\;+\;\beta 4_{i}{\text{TSR}}_{jk}^{2}$$

As we wished to generalize individual species’ responses based on their levels of urban sensitivity, we modeled all species‐specific parameters to derive from urban class‐specific hyperparameters for mean and precision (Ruiz‐Gutiérrez, Zipkin, & Dhondt, 2010). For example, if species *i* belonged to the “urban‐sensitive” class, the species‐specific parameter α1_*i*_ was modeled as α1_*i*_ ~ Normal(μ_α_sensitive_, σ_α_sensitive_). In this way, we estimated species‐specific parameters and their urban class‐specific hyperparameters simultaneously. Therefore, we can make direct statements about the probability distributions of any species‐specific parameter, or the urban class‐specific hyperparameter to which any group of species belonged. While such a priori hyperparameter groupings can affect individual parameter responses in the sampling process (Pacifici et al., 2014), we felt our approach was still beneficial because species‐specific parameter estimates are maintained, while allowing an analytical generalization to groups based on established criteria. We assumed that the surveyed populations were closed (no immigration or emigration) during the sampling period because our surveys were concentrated during a single breeding season. We acknowledge that by making this assumption, there is a chance of overestimating the abundance of cryptic species or species with low detection probabilities (Field et al., 2016). The prior specification for all mean (μ) hyperparameters was normally distributed and uninformative. The prior specification for all standard deviation (σ) hyperparameters was gamma distributed and uninformative.

We estimated bird species richness Richness_*jc*_ for each urban class *c* at each site *j* as the number of species belonging to each urban class at each site satisfying the logic *N*_*ij*_ ≥ 1. We similarly derived treatment and origin‐specific mean richness parameters for each urban class. For example:

(6) $$\mu {\text{Richness}}_{\mathrm{sensitive}\mathrm{grass}\mathrm{treated}}=\sum_{\begin{array}{c} \mathrm{ORI}[j]=\mathrm{grass}\\ \mathrm{TRT}[j]=\mathrm{treated}\\ c=\mathrm{sensitive} \end{array}}{\text{Richness}}_{jc}/n\left(j\right)$$

in the case of revegetated sites (treated open‐mowed grass) and urban sensitive species. We further derived parameters representing mean species richness benefit BRichness for each treatment for each urban class by subtracting the posterior distribution of the “untreated” mean species richness parameter from the “treated” species richness parameter. For example, BRichness_sensitive_grass_ = μRichness_sensitive_grass_treated_ − μRichness_sensitive_grass_untreated_. The structures of the variables TRT and ORI in equation (3) mean that we can use α3 and α4 as direct estimates of the benefit of treatments to bird abundances.

The species abundance model and the species richness model were processed using a Markov chain Monte Carlo simulation in the R package “rjags” (Plummer, 2016; R Core Team 2013). To test for the goodness of fit, we assessed trace plots and convergence statistics.

We express all results with a measure of central tendency and 95% credible interval. As a measure of significance, we calculated the proportion of the posterior distribution that fell above zero—this corresponds to the probability of the event occurring. The 95% credible intervals were calculated using the mode and the highest density interval (HDI) as this method creates the least amount of bias in assuming the shape of the distribution. The proportion of the posterior distribution greater than zero was calculated for each urban class and species parameter and used to express the effect of the restoration treatment.

## 3 Results

A total of 74 terrestrial bird species were observed during the survey period, all species were listed as common or least concern according to national and international threatened species legislation (Australian Government: Department of Environment and Energy 1999; IUCN 2017). Open‐mowed grass sites supported an estimated average of 11.93 species (*P* (BRichness) > 0 = 1), while revegetated sites supported an average of 19.91 species (*P* (BRichness) > 0 = 1); the benefit of revegetation was calculated to be an average increase of around 7.98 species (*P* (BRichness) > 0 = 1). Sites with weeds supported an estimated average of 20.1 species (*P* (BRichness) > 0 = 1), while weed controlled sites supported an average of 19.63 species (*P* (BRichness) > 0 = 1), suggesting that weed removal has little effect on species richness (*P* (BRichness) > 0 = 0.28). Output of species richness credible intervals can be found in the supplementary information. We did not find any results to suggest that patch area and treatment were confounded at the hyperparameter level (i.e., interaction terms were centered around zero, see supplementary information).

### 3.1 Urban class response

**Figure 2:**
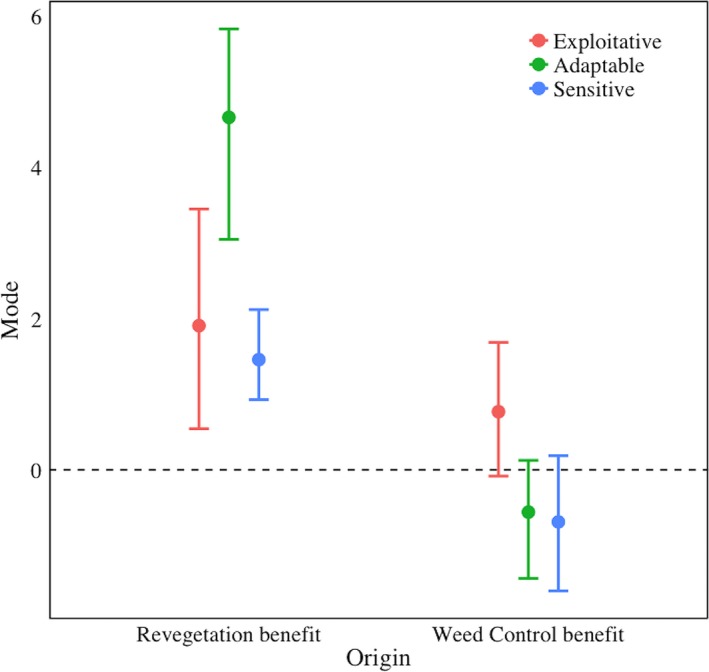
Modes and credible intervals of change in species richness of birds in the three urban classes for each restoration type—weed control (“Forest”) and revegetation (“Turf”). Values above the horizontal dashed line indicate positive responses to the treatment, and values below the line indicate negative responses

Average species richness increased for all three classes of urban bird species under the revegetation treatment. The greatest increase was seen in the urban adaptable class (4.67 species), with smaller increases in the urban exploitative (1.91 species) and urban sensitive (1.46 species) classes (Table 1, Figure 2).

**Table 1:** Hyperparameter highest credible intervals and proportion of distributions above zero

Parameter	HDI Lower	Mode	HDI Upper	Prop Above 0
$\mu \alpha 1_{\mathrm{exploiters}}$	−0.351	−0.14	0.091	0.11
$\mu \alpha 1_{\mathrm{adapters}}$	−0.499	−0.21	0.069	0.06
$\mu \alpha 1_{\mathrm{sensitive}}$	−0.907	−0.17	0.514	0.33
$\mu \alpha 2_{\mathrm{exploiters}}$	−0.181	0.04	0.277	0.64
$\mu \alpha 2_{\mathrm{adapters}}$	−0.415	−0.12	0.186	0.20
$\mu \alpha 2_{\mathrm{sensitive}}$	−1.098	−0.31	0.556	0.21
$\mu \alpha 3_{\mathrm{exploiters}}$	−1.484	0.70	3.014	0.65
$\mu \alpha 3_{\mathrm{adapters}}$	−4.360	−1.08	1.883	0.28
$\mu \alpha 3_{\mathrm{sensitive}}$	−5.537	−1.59	2.259	0.22
$\mu \alpha 4_{\mathrm{exploiters}}$	−0.807	1.62	4.123	0.86
$\mu \alpha 4_{\mathrm{adapters}}$	−1.173	2.17	5.121	0.88
$\mu \alpha 4_{\mathrm{sensitive}}$	−2.876	1.01	5.283	0.68

The effect of weed control on species richness varied with urban class. Species richness in the urban exploitative class was, on average, 0.77 species higher in nonweedy sites (*P* (BRichness) > 0 = 0.95). Whereas species richness was on average 0.56 species lower in nonweedy sites for urban adaptable (*P* (BRichness) > 0 = 0.07) and 0.69 species lower for urban sensitive (*P* (BRichness) > 0 = 0.07) classes (Table 1, Figure 2).

**Figure 3:**
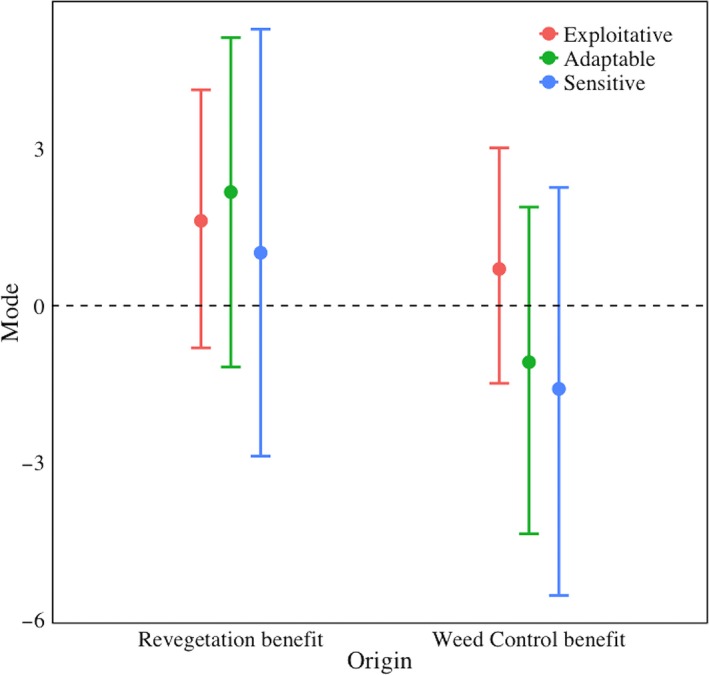
Modes and credible intervals of change abundance of birds in the three urban classes for each restoration type—weed control (“Forest”) and revegetation (“Turf”). Values above the horizontal dashed line indicate positive responses to the treatment, and values below the line indicate negative responses. The values displayed on the graph indicate the credible intervals of the posterior distribution of μ hyperparameters of α3 and α4 from equation (3)

There was no significant effect of revegetation or weed control on abundance of any urban class (Figure 3).

### 3.2 Individual species response

Sixty‐three of the 74 bird species (85%) show a > 50% probability of increasing in abundance in response to revegetation (Table S1). Urban exploitative species such as the sulfur crested cockatoo (*Cacatua galerita*), crested pigeon (*Ocyphaps lophotes*), rainbow lorikeet (*Trichoglossus moluccanus*), and Indian myna (*Acridotheres tristis*) all greatly increased in abundance within revegetated treatments (Table S1). Urban sensitive species predominantly increased in abundance within revegetated treatments, although a few species such as white‐throated gerygone (*Gerygone olivacea*), eastern yellow robin (*Eopsaltria australis*), and red‐browed finch (*Neochmia temporalis*) responded slightly less than 50% (Table S1).

Weed control presented mixed results at an individual species level. Twenty‐four of the 74 species detected increased in abundance are urban exploitative species, all of which (100%) increase in abundance between weedy and nonweedy sites (Table S2). Twenty‐one of the 74 species are classified as urban adaptable, and all species have a > 50% chance of declining in abundance in response to weed removal. Of the 29 species classified as urban sensitive, all have a > 50% chance of declining in response to weed removal, with some species such as the eastern yellow robin (*Eopsaltria australis*), eastern whipbird (*Psophodes olivaceus*), and rufous fantail (*Rhipidura rufifrons*) declining by >75%.

## 4 Discussion

Our results highlight the need to carefully consider conservation targets during planning and implementation of restoration activities, and the need to account for potentially perverse outcomes. Our study suggests that the probability of conservation benefits is much lower for weed management than for revegetation and that the risk of perverse outcome for bird species is more likely than not.

While revegetation of open grassy areas did increase the species richness of urban sensitive species, such as the tawny grassbird (*Megalurus timoriensis),* and spotted pardalote (*Pardalotus punctatus*), increases in species richness of urban exploitative and urban adaptable birds were higher. Furthermore, not only do urban sensitive species decrease in response to weed control, but urban adaptive species, such as noisy friarbird (*Philemon corniculatus*), silvereye (*Zosterops lateralis*), and laughing kookaburra (*Dacelo novaeguineae*), also increase. There were no urban exploitative species that decreased in response to weed control. These results support other studies from the region, which have found the presence of invasive plant species’ supports a greater species richness of small birds (Kath, Maron, & Dunn, 2009). This is likely due to the fact that removal of invasive plant species modifies the vegetation structure and causes the mid‐storey to become less structurally complex, potentially facilitating colonization by urban exploitative species such as noisy miners, which then suppress more sensitive birds species (Gosper & Vivian‐Smith, 2009; Hobbs, Higgs, & Harris, 2006; Hobbs et al., 2009). Furthermore, while all three urban classes increased, on average, in species richness when revegetation was implemented, urban adaptable species benefited most. This suggests that the ways in which we are currently revegetating urban green spaces favors species that are already more adaptable to urban landscapes. It is also important to note that while revegetated sites achieved the greatest increase in species richness, they had similar average species richness to the weed controlled and weedy sites. Therefore, while revegetation as an action may yield higher bird species richness benefit, revegetated sites as a greenspace may have no greater conservation value than forest fragments with or without weed control.

At a broader level, the quantity and the connectivity of greenspace within an urban matrix will have an important role in the amount of bird species the landscape can support (Fernández‐Juricic & Jokimäki, 2001). In Australia, large coordinated efforts have been designed and implemented with the specific goal of extending and connecting greenspaces within landscapes, for example, Gondwana Link, Habitat 141, Great Eastern Ranges (Gondwana Link Ltd 2015; Habitat141 2017; The Great Eastern Ranges Initiative 2017). But at a local level, the quality of urban greenspaces also impacts the abundance of bird species present within urban centers. Therefore, restoring urban greenspaces is an important component of conserving urban biodiversity by providing refugia, although currently we are not maximizing the conservation impact of these areas for species which rely on these areas the most (Fernández‐Juricic & Jokimäki, 2001; Shanahan, Miller, et al., 2011; Shanahan, Possingham, et al., 2011).

Restoring urban green spaces is expensive and therefore results such as those presented here have important implications for the planning of urban green space management. Information on the effectiveness of different actions is necessary for land managers to weigh up the costs and benefits prior to implementation. However, the decreases in urban sensitive species and increases in urban exploiters observed in response to weed control should not rule it out as a restoration action, but rather it should be implemented in concert with other management actions. For example, where weed control is necessary to meet other conservation objectives, urban sensitive bird species could benefit from an approach combining successional weed removal accompanied by native planting (Kath et al., 2009). Weed removal could also potentially lead to long‐term benefits by facilitating natural regeneration. While not considered here, the success of habitat restoration is another important factor in deciding which actions to implement, where and when. Restoration success can depend on many factors such as the management time‐frame, the long‐ and short‐term management goals as well as local and within site ecological variation, threat of re‐infestation, and other anthropogenic threats (Dearborn & Kark, 2010; Jellinek et al., 2014; Maas, Groenewegen, & Verheij, 2015).

Urban conservation is generally aiming to satisfy multiple conservation, recreational, and management goals. These actions not only contribute to securing urban biodiversity, but also to environmental education and human well‐being as many urban adaptable and sensitive bird species are also favorites among local people. For example, the superb fairywren (*Malurus* cyaneus), bush‐stone curlew (*Burhinus grallarius*), and spotted pardalote (*Pardalotus punctatus*) have been identified by the Australian public as their favorite birds (Birdlife 2016). The approach presented here, categorizing bird species into urban classes, can be used to understand how birds that are most at risk of being displaced from urban areas are responding to restoration actions, as an important step toward finding an optimal solution for the management of shared urban green spaces.

## Conflict of Interest

None declared.

## Data Accessibility

Data and scripts will be available through GitHub.

## Authors’ Contributions

CA, KM, DS, and HP conceived the ideas and designed the methodology; CA collected the data; CA and MM analyzed the data; DS contributed to framing the manuscript; CA led the writing of the manuscript. All authors contributed critically to the drafts and gave final approval for publication.
