# Supplementary material for: Assessing the impact of revegetation and weed control on urban sensitive bird species
Source: Ecol Evol. 2017 May 2;7(12):4200–8. doi: 10.1002/ece3.2960 (PMC5478067; doi:10.1002/ece3.2960)
Supplement: Supplementary file 1 [file ECE3-7-4200-s001.docx]

load("C:/Users/uqmmckin/Documents/CarlaUrbanBirdsAnalysis/carlaDat_2Dec.RData")

library(rjags)

## The data list

forjags<-list("cJ" = cJ,"cn" = cn,"cDATE"=cDATE,"cGRP"=cGRP,"cTRT"=cTRT,"cTSR"=cTSR,

"cTYPE"=cTYPE,"carea1"=carea1,"cy"=cy,"cK"=cK,"cURBE"=cURBE,"cURBA"=cURBA,

"cURBS"=cURBS,"cTRT.TYPE1"=cTRT.TYPE1,"cTRT.TYPE2"=cTRT.TYPE2,

"cCONT.TYPE1"=cCONT.TYPE1,"cCONT.TYPE2"= cCONT.TYPE2)

## parameters-to-keep list (short for simplicity)

parms<-c("mualpha1","mualpha2","mualpha3","mualpha4","muu1","muu2","muo1","muo2","alpha1", "alpha2", "alpha3","alpha4","cTRT.TYPE1.N.yearmean",

"cTRT.TYPE2.N.yearmean","cCONT.TYPE1.N.yearmean","cCONT.TYPE2.N.yearmean","cTRT.TYPE1.NcURBE.yearmean","cTRT.TYPE2.NcURBE.yearmean","cCONT.TYPE1.NcURBE.yearmean",

"cCONT.TYPE2.NcURBE.yearmean","cTRT.TYPE1.NcURBA.yearmean","cTRT.TYPE2.NcURBA.yearmean","cCONT.TYPE1.NcURBA.yearmean","cCONT.TYPE2.NcURBA.yearmean",

"cTRT.TYPE1.NcURBS.yearmean","cTRT.TYPE2.NcURBS.yearmean","cCONT.TYPE1.NcURBS.yearmean","cCONT.TYPE2.NcURBS.yearmean","u1","u2","o1","o2","muv1","mu.beta1",

"mu.beta2","mu.beta3","mu.beta4","v1","beta1","beta2","beta3","beta4")

##########################################

### Initiating values

## For model parameters

alpha1.init<-rnorm(cn)

alpha2.init<-rnorm(cn)

alpha3.init<-rnorm(cn)

alpha4.init<-rnorm(cn)

beta1.init<-rnorm(cn,mean=0,sd=0.0001)

beta2.init<-rnorm(cn,mean=0,sd=0.0001)

beta3.init<-rnorm(cn,mean=0,sd=0.0001)

beta4.init<-rnorm(cn,mean=0,sd=0.0001)

u1.init<-rnorm(cn,mean=0,sd=0.0001)

u2.init<-rnorm(cn,mean=0,sd=0.0001)

o1.init<-rnorm(cn,mean=0,sd=0.0001)

o2.init<-rnorm(cn,mean=0,sd=0.0001)

v1.init<-rnorm(cn,mean=0,sd=0.0001)

#### Prior means

muu1.init<-rnorm(3)

muu2.init<-rnorm(3)

muo1.init<-rnorm(3)

muo2.init<-rnorm(3)

mualpha1.init<-rnorm(3)

mualpha2.init<-rnorm(3)

mualpha3.init<-rnorm(3)

mualpha4.init<-rnorm(3)

muv1.init<-rnorm(3)

mu.beta1.init<-rnorm(3)

mu.beta2.init<-rnorm(3)

mu.beta3.init<-rnorm(3)

mu.beta4.init<-rnorm(3)

### For latent abundance

z.init<-cx

## Making inits for inclusion parameter w

w.init<-array(1,c(70,74,1)) # sites, species, year

### Bare minimum list of initial values

j.inits<-function(){list(alpha1=alpha1.init,alpha2=alpha2.init,alpha3=alpha3.init,alpha4=alpha4.init,mualpha1=mualpha1.init,mualpha2=mualpha2.init,

mualpha3=mualpha3.init,mualpha4=mualpha4.init,beta1=beta1.init,beta2=beta2.init,beta3=beta3.init,beta4=beta4.init,mu.beta1=mu.beta1.init,

mu.beta2=mu.beta2.init,mu.beta3=mu.beta3.init,mu.beta4=mu.beta4.init,Z=z.init,w=w.init,u1=u1.init,u2=u2.init,o1=o1.init,o2=o2.init,

muv1=muv1.init,v1=v1.init,muu1=muu1.init,muu2=muu2.init,muo1=muo1.init,muo2=muo2.init)}

cjags<-jags.model(file="C:/Users/uqmmckin/Documents/CarlaUrbanBirdsAnalysis/CarlaModel_4Sept.R",inits=j.inits,n.chains=3,n.adapt=10000,data=forjags)

update(cjags,n.iter=10000)

cjagsout<-coda.samples(cjags,n.iter=100000,thin=20,variable.names=parms)

summary(cjagsout)

plot(cjagsout)

save.image("C:/Users/uqmmckin/Documents/CarlaUrbanBirdsAnalysis/carlaMCMC2Dec.RData")
