# Supplementary material for: Assessing the impact of revegetation and weed control on urban sensitive bird species
Source: Ecol Evol. 2017 May 2;7(12):4200–8. doi: 10.1002/ece3.2960 (PMC5478067; doi:10.1002/ece3.2960)
Supplement: Supplementary file 2 [file ECE3-7-4200-s002.docx]

model {

# Priors

for (g in 1:3){ # Guilds: 1 = Urban Exploiters; 2 = Urban Adapterst; 3 = Urban Sensative

mualpha1[g] ~ dnorm(0,0.01)

mualpha2[g] ~ dnorm(0,0.01)

mualpha3[g] ~ dnorm(0,0.1)

mualpha4[g] ~ dnorm(0,0.1)

tau.alpha1[g] ~ dgamma(0.1,0.1)

tau.alpha2[g] ~ dgamma(0.1,0.1)

tau.alpha3[g] ~ dgamma(0.5,0.5)

tau.alpha4[g] ~ dgamma(0.5,0.5)

mu.beta1[g] ~ dnorm(0,0.0005)

mu.beta2[g] ~ dnorm(0,0.0005)

mu.beta3[g] ~ dnorm(0,0.0005)

mu.beta4[g] ~ dnorm(0,0.0005)

tau.beta1[g] ~ dgamma(0.1,0.1)

tau.beta2[g] ~ dgamma(0.1,0.1)

tau.beta3[g] ~ dgamma(0.1,0.1)

tau.beta4[g] ~ dgamma(0.1,0.1)

muu1[g] ~ dnorm(0,0.1)

muu2[g] ~ dnorm(0,0.1)

tau.u1[g] ~ dgamma(0.1,0.1)

tau.u2[g] ~ dgamma(0.1,0.1)

muo1[g] ~ dnorm(0,0.1)

muo2[g] ~ dnorm(0,0.1)

tau.o1[g] ~ dgamma(0.1,0.1)

tau.o2[g] ~ dgamma(0.1,0.1)

muv1[g] ~ dnorm(0,0.001)

tau.v1[g] ~ dgamma(0.1,0.1)

}

for(t in 1){

p.year[t] ~ dnorm(0,0.0001)

}

# Hyperparameters for imputing TSR (time) covariate (missing visits)

# mu.TSR ~ dnorm(0,0.001)

# tau.TSR ~ dgamma(0.001,0.001)

# for (j in 1:70){ # Site

# for (t in 1){ # Year

# for (k in 1:cK[j,t]){ # Survey

# cTSR[j,t,k] ~ dnorm(mu.TSR,tau.TSR)

#}}}

# Hyperparameters for imputing DATE covariate (missing visits)

#for (t in 1){ # Year

# for (k in 1:cK[1,t]){ # Survey

# mu.DATE[t,k] ~ dnorm(0,0.001)

#}}

# tau.DATE ~ dgamma(0.001,0.001)

# for (j in 1:cJ){ # Site

# for (t in 1){ # Year

# for (k in 1:cK[j,t]){ #Survey

# cDATE[j,t,k] ~ dnorm(mu.DATE[t,k],tau.DATE)

# }}}

## Urban Exploiting species modelling only

for(i in 1:24) {

# Covariates for Lambda

u1[i]~dnorm(muu1[cGRP[i]],tau.u1[cGRP[i]]) # Species abundance, Treated

u2[i]~dnorm(muu2[cGRP[i]],tau.u2[cGRP[i]]) # Species abundance, Untreated

o1[i] ~ dnorm(muo1[cGRP[i]], tau.o1[cGRP[i]]) # Origin Forest

o2[i] ~ dnorm(muo2[cGRP[i]], tau.o2[cGRP[i]]) # Origin Turf

alpha1[i] ~ dnorm(mualpha1[cGRP[i]], tau.alpha1[cGRP[i]]) # Patch Size (carea1)

alpha2[i] ~ dnorm(mualpha2[cGRP[i]], tau.alpha2[cGRP[i]]) # Treated * Patch Size interaction

alpha3[i] ~ dnorm(mualpha3[cGRP[i]], tau.alpha3[cGRP[i]]) # Treated * Forest Origin Interaction

alpha4[i] ~ dnorm(mualpha4[cGRP[i]], tau.alpha4[cGRP[i]]) # Treated * Turf Origin Interaction

psi[i] ~ dunif(0,1) # Suitability

# Covariates for p

v1[i]~dnorm(muv1[cGRP[i]],tau.v1[cGRP[i]]) # Species detectability

beta1[i]~dnorm(mu.beta1[cGRP[i]],tau.beta1[cGRP[i]]) # Date, linear

beta2[i]~dnorm(mu.beta2[cGRP[i]],tau.beta2[cGRP[i]]) # Date, quadratic

beta3[i]~dnorm(mu.beta3[cGRP[i]],tau.beta3[cGRP[i]]) # Time, linear

beta4[i]~dnorm(mu.beta4[cGRP[i]],tau.beta4[cGRP[i]]) # Time, quadratic

# Abundance model

for (t in 1){ # Year

for (j in 1:70) { # Site

log(lambda[j,i,t]) <- u1[i]*cTRT[j] + u2[i]*(1-cTRT[j]) +

o1[i]*cTYPE[j] + o2[i]*(1-cTYPE[j]) +

alpha1[i]*carea1[j] + alpha2[i]*carea1[j]*cTRT[j] +

alpha3[i]*cTRT[j]*cTYPE[j] + alpha4[i]*cTRT[j]*(1-cTYPE[j])

w[j,i,t] ~ dbern(psi[i]) # Estimate site suitability for ZIP

eff.lambda[j,i,t] <- w[j,i,t] * lambda[j,i,t] # ZIP

Z[j,i,t] ~ dpois(eff.lambda[j,i,t])

o[j,i,t] <- step(Z[j,i,t]-1) #

# Detection model

for (k in 1:cK[j,t]){ # Surveys

logit(p[j,i,t,k]) <- v1[i] + beta1[i]*cDATE[j,t,k] + beta2[i]*cDATE[j,t,k]*cDATE[j,t,k] +

beta3[i]*cTSR[j,t,k] + beta4[i]*cTSR[j,t,k]*cTSR[j,t,k]

##observed abundance as number of successes

cy[j,i,t,k] ~ dbin(p[j,i,t,k],Z[j,i,t])

}}}}

## For Urban Adapted species

for(i in 25:45) {

# Covariates for Lambda

u1[i]~dnorm(muu1[cGRP[i]],tau.u1[cGRP[i]]) # Species abundance, Treated

u2[i]~dnorm(muu2[cGRP[i]],tau.u2[cGRP[i]]) # Species abundance, Untreated

o1[i] ~ dnorm(muo1[cGRP[i]], tau.o1[cGRP[i]]) # Origin Forest

o2[i] ~ dnorm(muo2[cGRP[i]], tau.o2[cGRP[i]]) # Origin Turf

alpha1[i] ~ dnorm(mualpha1[cGRP[i]], tau.alpha1[cGRP[i]]) # Patch Size (carea1)

alpha2[i] ~ dnorm(mualpha2[cGRP[i]], tau.alpha2[cGRP[i]]) # Treated * Patch Size interaction

alpha3[i] ~ dnorm(mualpha3[cGRP[i]], tau.alpha3[cGRP[i]]) # Treated * Forest Origin Interaction

alpha4[i] ~ dnorm(mualpha4[cGRP[i]], tau.alpha4[cGRP[i]]) # Treated * Turf Origin Interaction

psi[i] ~ dunif(0,1) # Suitability

# Covariates for p

v1[i]~dnorm(muv1[cGRP[i]],tau.v1[cGRP[i]]) # Species detectability

beta1[i]~dnorm(mu.beta1[cGRP[i]],tau.beta1[cGRP[i]]) # Date, linear

beta2[i]~dnorm(mu.beta2[cGRP[i]],tau.beta2[cGRP[i]]) # Date, quadratic

beta3[i]~dnorm(mu.beta3[cGRP[i]],tau.beta3[cGRP[i]]) # Time, linear

beta4[i]~dnorm(mu.beta4[cGRP[i]],tau.beta4[cGRP[i]]) # Time, quadratic

# Abundance model

for (t in 1){ # Year

for (j in 1:70) { # Site

log(lambda[j,i,t]) <- u1[i]*cTRT[j] + u2[i]*(1-cTRT[j]) +

o1[i]*cTYPE[j] + o2[i]*(1-cTYPE[j]) +

alpha1[i]*carea1[j] + alpha2[i]*carea1[j]*cTRT[j] +

alpha3[i]*cTRT[j]*cTYPE[j] + alpha4[i]*cTRT[j]*(1-cTYPE[j])

w[j,i,t] ~ dbern(psi[i]) # Estimate site suitability for ZIP

eff.lambda[j,i,t] <- w[j,i,t] * lambda[j,i,t] # ZIP

Z[j,i,t] ~ dpois(eff.lambda[j,i,t])

o[j,i,t] <- step(Z[j,i,t]-1) # o = occupancy for i species (Z= latent abundance)

# Detection model

for (k in 1:cK[j,t]){ # Survey

logit(p[j,i,t,k]) <- v1[i] + beta1[i]*cDATE[j,t,k] + beta2[i]*cDATE[j,t,k]*cDATE[j,t,k] +

beta3[i]*cTSR[j,t,k] + beta4[i]*cTSR[j,t,k]*cTSR[j,t,k]

cy[j,i,t,k] ~ dbin(p[j,i,t,k],Z[j,i,t])

}}}}

## For Urban Sensative species

for(i in 46:74) {

# Covariates for Lambda

u1[i]~dnorm(muu1[cGRP[i]],tau.u1[cGRP[i]]) # Species abundance, Treated

u2[i]~dnorm(muu2[cGRP[i]],tau.u2[cGRP[i]]) # Species abundance, Untreated

o1[i] ~ dnorm(muo1[cGRP[i]], tau.o1[cGRP[i]]) # Origin Forest

o2[i] ~ dnorm(muo2[cGRP[i]], tau.o2[cGRP[i]]) # Origin Turf

alpha1[i] ~ dnorm(mualpha1[cGRP[i]], tau.alpha1[cGRP[i]]) # Patch Size (carea1)

alpha2[i] ~ dnorm(mualpha2[cGRP[i]], tau.alpha2[cGRP[i]]) # Treated * Patch Size interaction

alpha3[i] ~ dnorm(mualpha3[cGRP[i]], tau.alpha3[cGRP[i]]) # Treated * Forest Origin Interaction

alpha4[i] ~ dnorm(mualpha4[cGRP[i]], tau.alpha4[cGRP[i]]) # Treated * Turf Origin Interaction

psi[i] ~ dunif(0,1) # Suitability

# Covariates for p

v1[i]~dnorm(muv1[cGRP[i]],tau.v1[cGRP[i]]) # Species detectability

beta1[i]~dnorm(mu.beta1[cGRP[i]],tau.beta1[cGRP[i]]) # Date, linear

beta2[i]~dnorm(mu.beta2[cGRP[i]],tau.beta2[cGRP[i]]) # Date, quadratic

beta3[i]~dnorm(mu.beta3[cGRP[i]],tau.beta3[cGRP[i]]) # Time, linear

beta4[i]~dnorm(mu.beta4[cGRP[i]],tau.beta4[cGRP[i]]) # Time, quadratic

# Abundance model

for (t in 1){ # Year

for (j in 1:70) { # Site

log(lambda[j,i,t]) <- u1[i]*cTRT[j] + u2[i]*(1-cTRT[j]) +

o1[i]*cTYPE[j] + o2[i]*(1-cTYPE[j]) +

alpha1[i]*carea1[j] + alpha2[i]*carea1[j]*cTRT[j] +

alpha3[i]*cTRT[j]*cTYPE[j] + alpha4[i]*cTRT[j]*(1-cTYPE[j])

w[j,i,t] ~ dbern(psi[i]) # Estimate site suitability for ZIP

eff.lambda[j,i,t] <- w[j,i,t] * lambda[j,i,t] # ZIP

Z[j,i,t] ~ dpois(eff.lambda[j,i,t])

o[j,i,t] <- step(Z[j,i,t]-1) #

# Detection model

for (k in 1:cK[j,t]){ # Survey

logit(p[j,i,t,k]) <- v1[i] + beta1[i]*cDATE[j,t,k] + beta2[i]*cDATE[j,t,k]*cDATE[j,t,k] +

beta3[i]*cTSR[j,t,k] + beta4[i]*cTSR[j,t,k]*cTSR[j,t,k]

cy[j,i,t,k] ~ dbin(p[j,i,t,k],Z[j,i,t])

}}}}

# Species Richness model

## Derived values of richness by guild, treatment, and origin (Turf or Forest)

for(t in 1){ # Year

for(j in 1:cJ){ # Site

cNsite[j,t]<- sum(o[j,,t]) # Total richness for the site

NcURBE[j,t]<- inprod(o[j,,t], cURBE[]) # Richness, urban exploiters

NcURBA[j,t]<- inprod(o[j,,t],cURBA[]) # Richness, urban adapters

NcURBS[j,t]<- inprod(o[j,,t],cURBS[]) # Richness, urban sensative

cTRT.N[j,t]<-cNsite[j,t]*cTRT[j] # Richness, treated (revegetation or weed control)

cCONT.N[j,t]<-cNsite[j,t]*(1-cTRT[j]) # Richness, not treated (not revegetation and not weed controled)

cTRT.NcURBE[j,t]<-NcURBE[j,t]*cTRT[j] # Species richness of URBE in treated sites

cCONT.NcURBE[j,t]<-NcURBE[j,t]*(1-cTRT[j]) # Species richness of URBE in untreated sites

cTRT.NcURBA[j,t]<-NcURBA[j,t]*cTRT[j] # Species richness of URBA in treated sites

cCONT.NcURBA[j,t]<-NcURBA[j,t]*(1-cTRT[j]) # Species richness of URBA in untreated sites

cTRT.NcURBS[j,t]<-NcURBS[j,t]*cTRT[j] # Species richness of URBS in treated sites

cCONT.NcURBS[j,t]<-NcURBS[j,t]*(1-cTRT[j]) # Species richness of URBS in untreated sites

}

# Mean richness by block and corridor sites for treatment and control

cTRT.TYPE1.N.yearmean[t]<-inprod(cNsite[,t],cTRT.TYPE1[])/16 # Treated Forest (WA)

cTRT.TYPE2.N.yearmean[t]<-inprod(cNsite[,t],cTRT.TYPE2[])/19 # Treated Turf (RV)

cCONT.TYPE1.N.yearmean[t]<-inprod(cNsite[,t],cCONT.TYPE1[])/16 # Control Forest (WP)

cCONT.TYPE2.N.yearmean[t]<-inprod(cNsite[,t],cCONT.TYPE2[])/19 # ControlTurf (TF)

cTRTben.forest.N.yearmean[t]<-(cTRT.TYPE1.N.yearmean[t])-(cCONT.TYPE1.N.yearmean[t]) ##net benefit in forest

cTRTben.turf.N.yearmean[t]<-(cTRT.TYPE2.N.yearmean[t])-(cCONT.TYPE2.N.yearmean[t]) #net benefit turf

cTRT.TYPE1.NcURBE.yearmean[t]<-inprod(NcURBE[,t],cTRT.TYPE1[])/16

cTRT.TYPE2.NcURBE.yearmean[t]<-inprod(NcURBE[,t],cTRT.TYPE2[])/19

cCONT.TYPE1.NcURBE.yearmean[t]<-inprod(NcURBE[,t],cCONT.TYPE1[])/16

cCONT.TYPE2.NcURBE.yearmean[t]<-inprod(NcURBE[,t],cCONT.TYPE2[])/19

cTRTben.forest.NcURBE.yearmean[t]<-(cTRT.TYPE1.NcURBE.yearmean[t])-(cCONT.TYPE1.NcURBE.yearmean[t]) ##net benefit in forest URBE

cTRTben.turf.NcURBE.yearmean[t]<-(cTRT.TYPE2.NcURBE.yearmean[t])-(cCONT.TYPE2.NcURBE.yearmean[t]) #net benefit turf URBE

cTRT.TYPE1.NcURBA.yearmean[t]<-inprod(NcURBA[,t],cTRT.TYPE1[])/16

cTRT.TYPE2.NcURBA.yearmean[t]<-inprod(NcURBA[,t],cTRT.TYPE2[])/19

cCONT.TYPE1.NcURBA.yearmean[t]<-inprod(NcURBA[,t],cCONT.TYPE1[])/16

cCONT.TYPE2.NcURBA.yearmean[t]<-inprod(NcURBA[,t],cCONT.TYPE2[])/19

cTRTben.forest.NcURBA.yearmean[t]<-(cTRT.TYPE1.NcURBA.yearmean[t])-(cCONT.TYPE1.NcURBA.yearmean[t]) ##net benefit in forest URBA

cTRTben.turf.NcURBA.yearmean[t]<-(cTRT.TYPE2.NcURBA.yearmean[t])-(cCONT.TYPE2.NcURBA.yearmean[t]) #net benefit turf URBA

cTRT.TYPE1.NcURBS.yearmean[t]<-inprod(NcURBS[,t],cTRT.TYPE1[])/16

cTRT.TYPE2.NcURBS.yearmean[t]<-inprod(NcURBS[,t],cTRT.TYPE2[])/19

cCONT.TYPE1.NcURBS.yearmean[t]<-inprod(NcURBS[,t],cCONT.TYPE1[])/16

cCONT.TYPE2.NcURBS.yearmean[t]<-inprod(NcURBS[,t],cCONT.TYPE2[])/19

cTRTben.forest.NcURBS.yearmean[t]<-(cTRT.TYPE1.NcURBS.yearmean[t])-(cCONT.TYPE1.NcURBS.yearmean[t]) ##net benefit in forest URBS

cTRTben.turf.NcURBS.yearmean[t]<-(cTRT.TYPE2.NcURBS.yearmean[t])-(cCONT.TYPE2.NcURBS.yearmean[t]) #net benefit turf URBS

}

## Computation of fit statistic

# Fit statistic for observed data

for(j in 1:cJ){ # Site

for(i in 1:cn){ # Species

for(t in 1){ # Year

for(k in 1:cK[j,t]){ # Survey

eval[j,i,t,k]<-p[j,i,t,k]*Z[j,i,t] # Expected value ### Changed N to Z ###

E[j,i,t,k] <- pow((cy[j,i,t,k] - eval[j,i,t,k]),2) / (eval[j,i,t,k] + 0.5) # Chi square-type discrepancy

}

dsum[j,i,t] <- sum(E[j,i,t,1:cK[j,t]])

}}}

fit <- sum(dsum[,,]) # Sum up for actual data set

# Generate replicate data and compute fit stats for them

for (j in 1:cJ){ # Site

for (i in 1:cn){ # Species

for (t in 1){ # Year

for (k in 1:cK[j,t]){ # Survey

C.new[j,i,t,k]~dbin(p[j,i,t,k],Z[j,i,t]) ### Changed N to Z ###

E.new[j,i,t,k] <- pow((C.new[j,i,t,k] - eval[j,i,t,k]),2) / (eval[j,i,t,k] + 0.5)

} # k

dnewsum[j,i,t]<-sum(E.new[j,i,t,1:cK[j,t]])

}}}

fit.new <- sum(dnewsum[,,])

bpvalue<-step(fit.new-fit) # Bayesian P-value for goodness-of-fit test

} # End model code
