# Supplementary material for: Assessing the impact of revegetation and weed control on urban sensitive bird species
Source: Ecol Evol. 2017 May 2;7(12):4200–8. doi: 10.1002/ece3.2960 (PMC5478067; doi:10.1002/ece3.2960)
Supplement: Supplementary file 3 [file ECE3-7-4200-s003.docx]

**Supporting Information**

Table S1Credible intervals and probability of bird species benefiting from revegetation (*PropAbove0:* the probability above 0 indicates the likelihood of the species population increasing in population size from the treatment). The table is ordered from species that increase in abundance most to those that increase least. Species above the horizontal line benefit more than 70%.

| **Species (Common Name)** | **Urban Class** | **HDI Lower** | **Mean** | **HDI Upper** | **PropAbove0** |
| --- | --- | --- | --- | --- | --- |
| Sulphur Crested Cockatoo | Exploitative | 2.943 | 5.564 | 8.155 | 1.000 |
| Crested Pigeon | Exploitative | 1.339 | 4.056 | 6.844 | 0.992 |
| Indian Myna | Exploitative | 1.089 | 3.815 | 6.780 | 0.989 |
| Rock Dove | Exploitative | 0.939 | 3.702 | 6.486 | 0.988 |
| Tawny Grassbird | Sensitive | 0.034 | 5.406 | 10.361 | 0.979 |
| Laughing Kookaburra | Adaptable | 0.048 | 3.518 | 6.786 | 0.972 |
| Spotted Pardalote | Sensitive | -0.055 | 5.273 | 10.730 | 0.972 |
| Eastern Koel | Exploitative | 0.175 | 3.076 | 6.259 | 0.960 |
| Little Friarbird | Sensitive | -0.739 | 4.297 | 9.417 | 0.953 |
| White-browed Scrubwren | Adaptable | -0.597 | 2.959 | 6.245 | 0.944 |
| Rainbow Lorikeet | Exploitative | -0.141 | 2.303 | 4.952 | 0.932 |
| Spangled Drongo | Adaptable | -0.629 | 2.831 | 6.177 | 0.930 |
| Pheasant Coucal | Adaptable | -0.992 | 2.729 | 6.021 | 0.922 |
| Lewin’s Honeyeater | Adaptable | -0.991 | 2.670 | 6.050 | 0.919 |
| Scaly-breasted Lorikeet | Exploitative | -0.592 | 2.603 | 6.083 | 0.916 |
| Forest Kingfisher | Sensitive | -1.792 | 3.386 | 8.979 | 0.899 |
| Masked Lapwing | Exploitative | -0.365 | 1.924 | 4.379 | 0.896 |
| Brown Quail | Adaptable | -1.190 | 2.393 | 5.705 | 0.892 |
| Channel-billed Cuckoo | Adaptable | -1.199 | 2.277 | 5.508 | 0.890 |
| Striated Pardalote | Adaptable | -1.351 | 2.340 | 5.694 | 0.886 |
| Noisy Friarbird | Adaptable | -1.350 | 2.314 | 5.631 | 0.884 |
| Peaceful Dove | Sensitive | -1.863 | 2.922 | 7.876 | 0.883 |
| Magpie Lark | Exploitative | -1.259 | 2.370 | 6.558 | 0.878 |
| Welcome Swallow | Exploitative | -0.819 | 1.807 | 4.519 | 0.871 |
| Blue-faced Honeyeater | Adaptable | -1.463 | 2.167 | 5.449 | 0.871 |
| King Parrot | Adaptable | -1.409 | 2.171 | 5.559 | 0.870 |
| Willie Wagtail | Exploitative | -0.811 | 1.781 | 4.422 | 0.865 |
| Pale-headed Rosella | Adaptable | -1.538 | 2.114 | 5.500 | 0.859 |
| Olive-backed Oriole | Adaptable | -1.610 | 2.018 | 5.473 | 0.851 |
| Sacred Kingfisher | Adaptable | -1.812 | 2.040 | 5.651 | 0.842 |
| Silvereye | Adaptable | -2.092 | 2.006 | 5.658 | 0.835 |
| Little Shrike-thrush | Sensitive | -2.500 | 2.113 | 6.788 | 0.816 |
| Black-faced Cuckoo-shrike | Exploitative | -1.148 | 1.443 | 4.041 | 0.812 |
| Bar-shouldered Dove | Adaptable | -2.172 | 1.788 | 5.611 | 0.806 |
| Dollarbird | Adaptable | -2.104 | 1.630 | 4.953 | 0.800 |
| Pacific Baza | Adaptable | -2.272 | 1.533 | 4.935 | 0.785 |
| Superb Fairywren | Adaptable | -2.568 | 1.553 | 5.413 | 0.771 |
| Australian Brush Turkey | Adaptable | -2.153 | 1.366 | 4.689 | 0.762 |
| Brown Honeyeater | Adaptable | -2.628 | 1.315 | 4.785 | 0.748 |
| Grey Shrike-thrush | Sensitive | -3.621 | 1.602 | 6.642 | 0.729 |
| Pied Currawong | Exploitative | -1.564 | 1.031 | 3.827 | 0.727 |
| Eastern Whipbird | Sensitive | -3.601 | 1.546 | 6.740 | 0.725 |
| Collared Sparrowhawk | Sensitive | -3.623 | 1.535 | 6.558 | 0.723 |
| Short-billed Corella | Exploitative | -1.699 | 1.017 | 3.842 | 0.714 |
| Striated Thornbill | Sensitive | -3.689 | 1.390 | 6.488 | 0.704 |
| Figbird | Exploitative | -3.742 | 1.328 | 6.402 | 0.696 |
| Rainbow Bee-eater | Sensitive | -3.259 | 1.079 | 5.748 | 0.677 |
| White-breasted Woodswallow | Exploitative | -4.230 | 1.171 | 6.332 | 0.674 |
| Red-backed Fairywren | Sensitive | -4.044 | 1.121 | 6.025 | 0.667 |
| Torresian Crow | Exploitative | -4.139 | 0.787 | 5.382 | 0.625 |
| Pied Butcherbird | Exploitative | -4.842 | 0.739 | 5.899 | 0.607 |
| Spotted Dove | Exploitative | -5.353 | 0.518 | 6.156 | 0.580 |
| Golden Whistler | Sensitive | -7.015 | 0.640 | 8.198 | 0.565 |
| Rufous Fantail | Sensitive | -6.515 | 0.317 | 6.911 | 0.557 |
| Noisy Miner | Exploitative | -2.713 | 0.261 | 3.471 | 0.556 |
| Fantailed Cuckoo | Sensitive | -6.575 | 0.160 | 6.978 | 0.544 |
| Mangrove Gerygone | Sensitive | -6.895 | 0.196 | 6.838 | 0.538 |
| Golden-headed Cisticola | Sensitive | -6.660 | 0.148 | 6.934 | 0.534 |
| Apostlebird | Sensitive | -6.660 | 0.118 | 6.826 | 0.529 |
| Rufous Whistler | Sensitive | -6.868 | 0.053 | 6.638 | 0.522 |
| Black-faced Monarch | Sensitive | -6.948 | -0.033 | 6.719 | 0.515 |
| Mistletoebird | Sensitive | -7.031 | -0.067 | 6.275 | 0.511 |
| Variegated Fairywren | Sensitive | -6.617 | -0.083 | 6.557 | 0.505 |
| White-throated Gerygone | Sensitive | -6.696 | -0.165 | 6.552 | 0.498 |
| Eastern Yellow Robin | Sensitive | -6.748 | -0.210 | 6.266 | 0.497 |
| Red-browed Finch | Sensitive | -6.811 | -0.224 | 6.248 | 0.490 |
| Leaden Flycatcher | Sensitive | -6.981 | -0.227 | 6.187 | 0.485 |
| Australian Reed Warbler | Sensitive | -7.198 | -0.476 | 5.989 | 0.460 |
| Varied Sittella | Sensitive | -7.428 | -0.521 | 5.568 | 0.453 |
| Galah | Exploitative | -2.964 | -0.235 | 2.553 | 0.446 |
| Australian Magpie | Exploitative | -2.749 | -0.261 | 2.450 | 0.439 |
| Fairy Martin | Exploitative | -2.749 | -0.270 | 2.275 | 0.437 |
| Scarlet Honeyeater | Sensitive | -7.761 | -1.210 | 4.948 | 0.366 |
| Grey Butcherbird | Exploitative | -4.433 | -1.124 | 2.482 | 0.288 |

Table S2 Credible intervals and probability of bird species benefiting from weed control (*PropAbove0:* the probability above 0 indicates the likelihood of the species population increasing in population size from the treatment). The table is ordered from species that increase in abundance most to those that increase least.

| **Species** | **Urban Class** | **HDI Lower** | **Mean** | **HDI Upper** | **PropAbove0** |
| --- | --- | --- | --- | --- | --- |
| Galah | Exploitative | -1.455 | 0.968 | 3.577 | 0.717 |
| Australian Magpie | Exploitative | -1.492 | 0.912 | 3.439 | 0.707 |
| Eastern Koel | Exploitative | -1.484 | 0.845 | 3.329 | 0.697 |
| Pied Currawong | Exploitative | -1.512 | 0.852 | 3.382 | 0.696 |
| Masked Lapwing | Exploitative | -1.450 | 0.863 | 3.257 | 0.695 |
| Scaly Breasted Lorikeet | Exploitative | -1.566 | 0.814 | 3.417 | 0.686 |
| Noisy Miner | Exploitative | -1.673 | 0.788 | 3.451 | 0.677 |
| Rainbow Lorikeet | Exploitative | -1.539 | 0.779 | 3.293 | 0.673 |
| Rock Dove | Exploitative | -1.670 | 0.733 | 3.178 | 0.665 |
| Fairy Martin | Exploitative | -1.768 | 0.708 | 3.162 | 0.659 |
| Figbird | Exploitative | -1.773 | 0.687 | 3.388 | 0.654 |
| Torresian Crow | Exploitative | -1.776 | 0.677 | 3.373 | 0.654 |
| Crested Pigeon | Exploitative | -1.611 | 0.704 | 3.240 | 0.650 |
| White-breasted Woodswallow | Exploitative | -1.808 | 0.671 | 3.327 | 0.649 |
| Magpie Lark | Exploitative | -1.775 | 0.641 | 3.298 | 0.644 |
| Pied Butcherbird | Exploitative | -1.918 | 0.629 | 3.276 | 0.643 |
| Welcome Swallow | Exploitative | -1.725 | 0.619 | 3.165 | 0.642 |
| Indian Myna | Exploitative | -1.672 | 0.638 | 3.157 | 0.641 |
| Black-faced Cuckooshrike | Exploitative | -1.763 | 0.615 | 3.151 | 0.635 |
| Spotted Dove | Exploitative | -1.967 | 0.578 | 3.233 | 0.629 |
| Willy Wagtail | Exploitative | -1.817 | 0.568 | 3.104 | 0.624 |
| Grey Butcherbird | Exploitative | -1.983 | 0.542 | 3.181 | 0.623 |
| Sulphur Crested Cockatoo | Exploitative | -1.633 | 0.539 | 3.032 | 0.611 |
| Short-billed Corella | Exploitative | -2.237 | 0.325 | 3.033 | 0.572 |
| Olive-backed Oriole | Adaptable | -4.233 | -0.545 | 2.663 | 0.393 |
| Australian Brush Turkey | Adaptable | -4.167 | -0.666 | 2.432 | 0.365 |
| Pacific Baza | Adaptable | -4.361 | -0.712 | 2.554 | 0.359 |
| Bar-shouldered Dove | Adaptable | -4.295 | -0.722 | 2.568 | 0.355 |
| Pale-headed Rosella | Adaptable | -4.378 | -0.795 | 2.380 | 0.341 |
| Varied Sittella | Sensitive | -5.177 | -0.971 | 3.111 | 0.331 |
| Channel-billed Cuckoo | Adaptable | -4.319 | -0.854 | 2.186 | 0.326 |
| Lewins Honeyeater | Adaptable | -4.384 | -0.894 | 2.193 | 0.317 |
| King Parrot | Adaptable | -4.509 | -0.913 | 2.064 | 0.316 |
| Striated Pardalote | Adaptable | -4.538 | -1.020 | 2.245 | 0.302 |
| Brown Honeyeater | Adaptable | -4.584 | -1.009 | 2.128 | 0.300 |
| Dollarbird | Adaptable | -4.589 | -1.061 | 2.215 | 0.293 |
| Mangrove Gerygone | Sensitive | -5.339 | -1.200 | 3.060 | 0.293 |
| Little Friarbird | Sensitive | -5.358 | -1.218 | 2.909 | 0.290 |
| Blue-faced Honeyeater | Adaptable | -4.602 | -1.139 | 2.056 | 0.278 |
| Golden Whistler | Sensitive | -5.533 | -1.290 | 2.847 | 0.276 |
| Apostlebird | Sensitive | -5.521 | -1.349 | 2.762 | 0.271 |
| Sacred Kingfisher | Adaptable | -4.825 | -1.215 | 2.300 | 0.271 |
| Fantailed Cuckoo | Sensitive | -5.475 | -1.369 | 2.853 | 0.267 |
| Brown Quail | Adaptable | -4.666 | -1.220 | 1.947 | 0.264 |
| Little Shrike Thrush | Sensitive | -5.466 | -1.412 | 2.751 | 0.259 |
| White-browed Scrubwren | Adaptable | -4.682 | -1.256 | 1.879 | 0.259 |
| Australian Reed Warbler | Sensitive | -5.605 | -1.410 | 2.732 | 0.257 |
| Tawny Grassbird | Sensitive | -5.538 | -1.454 | 2.661 | 0.254 |
| Superb Fairywren | Adaptable | -4.880 | -1.374 | 2.105 | 0.247 |
| Rainbow Bee-eater | Sensitive | -5.649 | -1.462 | 2.556 | 0.244 |
| Spangled Drongo | Adaptable | -4.751 | -1.317 | 1.775 | 0.244 |
| Leaden Flycatcher | Sensitive | -5.712 | -1.521 | 2.648 | 0.243 |
| Laughing Kookaburra | Adaptable | -4.709 | -1.344 | 1.737 | 0.242 |
| Scarlet Honeyeater | Sensitive | -5.589 | -1.540 | 2.733 | 0.239 |
| Pheasant Coucal | Adaptable | -4.919 | -1.454 | 1.868 | 0.228 |
| Peaceful Dove | Sensitive | -5.723 | -1.650 | 2.678 | 0.225 |
| Red-backed Fairywren | Sensitive | -5.831 | -1.668 | 2.575 | 0.225 |
| Silvereye | Adaptable | -5.126 | -1.503 | 1.830 | 0.225 |
| Striated Thornbil | Sensitive | -5.932 | -1.661 | 2.387 | 0.224 |
| Spotted Pardalote | Sensitive | -5.864 | -1.695 | 2.477 | 0.222 |
| Rufous Whistler | Sensitive | -5.791 | -1.667 | 2.463 | 0.221 |
| Red-browed Finch | Sensitive | -5.921 | -1.670 | 2.476 | 0.221 |
| Forest Kingfisher | Sensitive | -5.913 | -1.754 | 2.531 | 0.218 |
| Golden-headed Cisticola | Sensitive | -6.046 | -1.766 | 2.541 | 0.215 |
| Grey Shrike-thrush | Sensitive | -6.009 | -1.762 | 2.450 | 0.214 |
| Variegated Fairywren | Sensitive | -6.101 | -1.785 | 2.482 | 0.214 |
| Rufous Fantail | Sensitive | -5.937 | -1.780 | 2.546 | 0.211 |
| Mistletoebird | Sensitive | -5.932 | -1.741 | 2.408 | 0.210 |
| Black-faced Monarch | Sensitive | -6.003 | -1.832 | 2.390 | 0.210 |
| Collared Sparrowhawk | Sensitive | -5.930 | -1.782 | 2.551 | 0.209 |
| White-throated Gerygone | Sensitive | -6.004 | -1.816 | 2.451 | 0.205 |
| Eastern Whipbird | Sensitive | -6.162 | -1.894 | 2.332 | 0.194 |
| Noisy Friarbird | Adaptable | -5.239 | -1.703 | 1.641 | 0.193 |
| Eastern Yellow Robin | Sensitive | -6.261 | -2.037 | 2.406 | 0.184 |
